# Whole-Genome Analysis of the Cell Cycle Regulators in Soybean: Evolution, Expansion, and Functional Implications

**DOI:** 10.3390/biology15131065

**Published:** 2026-07-03

**Authors:** Qianru Jia, Jinghui Shi, Rui Wang, Xiaoqi He, Binhui Guo, Guanglong Zhu, Li Song

**Affiliations:** 1 Joint International Research Laboratory of Agriculture and Agri-Product Safety, Institutes of Agricultural Science and Technology Development, Yangzhou University, Yangzhou 225009, China; 008967@yzu.edu.cn (Q.J.); 18035610914@163.com (J.S.); 18168627603@163.com (R.W.); hx19416187@163.com (X.H.); bhguo@yzu.edu.cn (B.G.); 2 Zhongshan Biological Breeding Laboratory, Yangzhou University, Yangzhou 225009, China

**Keywords:** cell cycle, cyclin-dependent kinases, cyclins, soybean, complexes, gene expression, gene duplication, seed development

## Abstract

Cyclin-dependent kinases (CDKs) and cyclins are master regulators of the cell cycle, playing critical roles in plant growth, development, and stress responses. While these gene families have been extensively studied in model plants, a comprehensive analysis in soybean remains underexplored. To address this gap, we performed a genome-wide identification and systematic analysis of these families in soybean using bioinformatic approaches. Expression profiles and protein interactions of selected *GmCDK* and *GmCyclin* candidates were tested by qRT-PCR and BiFC assays. A total of 28 *GmCDK* and 101 *GmCyclin* genes were identified, revealing a significant expansion compared to *Arabidopsis*, rice, and maize, primarily driven by whole-genome and segmental duplications. Phylogenetic analysis classified *GmCDKs* into seven conserved clades (CDKA-CDKG) and *GmCyclins* into ten distinct subfamilies. Expression profiling demonstrated dynamic, tissue-specific patterns, with distinct modules active during seed development and in tissues. Promoter analysis further linked these genes to hormonal and stress-responsive pathways. Crucially, BiFC assay identified specific interactions between GmCDKA2, GmCDKA3, GmCDKB1 and GmCYCA3-3, suggesting evolutionary divergence in soybean CDK-Cyclin regulatory networks. This study provides a foundational resource for the soybean cell cycle regulome, highlighting its evolutionary plasticity and implicating specific CDK-Cyclin pairs as potential targets for manipulating agronomic traits such as seed development and stress resilience.

## Simple Summary

Plant growth and development rely on precisely controlled cell division, which is governed by cyclin-dependent kinases and Cyclin genes. Soybean is a globally important crop, yet its cell division control genes remain poorly studied. In this study, we identified the cyclin-dependent kinases and Cyclin gene families in the soybean genome and revealed a notable expansion compared to other plants. These genes exhibit distinct expression patterns across various tissues and developmental stages, particularly during seed formation. Protein interaction assays confirmed functional associations among specific cyclin-dependent kinases and cyclin pairs. Our findings establish a foundational genetic resource for soybean improvement, with potential applications in enhancing seed development and environmental resilience.

## 1. Introduction

The precise regulation of the cell cycle is fundamental to plant growth, organogenesis, and environmental adaptation. This process is governed by a core regulatory network centered on the coordinated action of cyclin-dependent kinases (CDKs) and cyclins [1]. As serine/threonine kinases, CDKs must bind to cyclins to form active complexes. These complexes drive key cell cycle transitions—from G1 to S and G2 to M—by phosphorylating downstream substrates such as the retinoblastoma-related protein (RBR) and histone H1 [2,3].

In plants, the CDK family is divided into seven subfamilies (CDKA–CDKG). CDKA, containing a conserved PSTAIRE motif, regulates the G1/S transition, while the plant-specific CDKB governs G2/M progression and mitosis. The remaining subfamilies (CDKC–G) are involved in non-canonical functions, including transcriptional regulation, meiosis, and stress responses [4,5]. Correspondingly, the plant cyclin family normally comprises ten subclasses (A, B, C, D, H, J, L, P, T, and SDS). A- and B-type cyclins regulate S-phase progression and M-phase execution, respectively. D-type cyclins act as “signal integrators”, initiating the G1/S transition in response to hormones such as cytokinins and auxins. Plant-specific P-type cyclins (the U subfamily) enable cell cycle adaptation to nutrient signals, including phosphate starvation [6,7].

Beyond their canonical roles in cell cycle progression, these proteins are increasingly recognized as key integrators of developmental and environmental signals. In *Arabidopsis*, CDKC2 regulates far-red light-mediated hypocotyl elongation via histone deacetylation [8], while the CDKG1/CYCLINL complex ensures proper male meiosis through mRNA processing [9]. Overexpression of the cotton *CDKF4* gene in *Arabidopsis thaliana* can significantly enhance its drought and salt tolerance [10]. In rice, specific cyclins directly influence agronomic traits, such as *OsCycC* in grain size [11], *OsCYCU4* in leaf angle [12], and *OsCYCP4;4* in nutrient stress adaption [13]. This functional diversity is further illustrated by CYCB1’s role in stomatal division and growth-defense coordination in *Arabidopsis* [14,15].

Plant cell cycle regulation is uniquely adapted to sessile growth, exhibiting high plasticity and sensitivity to environmental cues such as light, hormones, and nutrients. Consequently, CDK–Cyclin networks are deeply embedded in stress-response and developmental pathways. In *Arabidopsis*, CDK8 promotes salt tolerance by degrading a transcriptional repressor and regulates lipid synthesis [3,16]. CDKG2 negatively regulates salinity responses and flowering time [2], and CDKC;2 coordinates cell division and organ size with drought tolerance through transcriptional regulation via phosphorylation of RNA polymerase II [17]. Together, these findings underscore the functional diversification of CDK–Cyclin modules as central hubs connecting cell cycle control with developmental patterning and environmental adaptation.

Soybean (*Glycine max*) is a globally critical source of edible oil and plant protein, serving as a cornerstone for food security and sustainable agriculture [18]. Its agronomic value is largely determined by yield-related traits such as seed size, seed weight, and plant architecture, all of which are intrinsically linked to cell division and organ growth. Furthermore, soybean possesses a unique ability to form symbiotic nitrogen-fixing nodules with rhizobia, a process that requires precisely coordinated cell proliferation in the root cortex and pericycle [19]. Given the sessile nature of plants, soybean growth and yield are also frequently constrained by abiotic stresses like drought and salinity, which trigger cell cycle arrest as part of adaptive responses. Therefore, unraveling the regulatory mechanisms of the cell cycle in soybean is of paramount importance for the genetic improvement of both yield potential and stress resilience.

While the roles of CDK and CYC have been systematically characterized in a range of crops, including rice [20], maize [21], rapeseed [22], cotton [10], peanut [22] and quinoa [23], significant knowledge gaps persist for soybean (*Glycine max*). As a paleopolyploid species, soybean has experienced two rounds of whole-genome duplication, resulting in a large and complex gene family that may exhibit functional redundancy or neofunctionalization compared to their orthologs in *Arabidopsis* or rice [20]. To date, only a few members (e.g., GmCDKA;1) have been preliminarily described, and a comprehensive genome-wide identification of the entire CDK and cyclin families—including their evolutionary relationships, duplication patterns, and tissue-specific expression profiles during nodule development and seed formation—is still lacking.

In this study, we performed a systematic identification and comprehensive analysis of *CDK* and *Cyclin* gene families in the soybean genome, including phylogenetic relationships, chromosomal locations, evolutionary patterns, and expression characteristics. Our findings provide a theoretical basis for elucidating the unique mechanisms of cell cycle regulation in legumes and offer valuable candidate targets for the genetic improvement of important agronomic traits in soybean.

## 2. Materials and Methods

### 2.1. Identification of GmCDK and GmCyclin Genes in Glycine Max

Reference protein sequences of *CDKs* and *Cyclins* from *Arabidopsis thaliana* were retrieved from the TAIR database (https://www.arabidopsis.org/ (accessed on 20 November 2025)) and employed as queries for homology-based searches. Soybean protein sequences and corresponding genome annotations (assembly Wm82.a6.v1) were obtained from Phytozome (https://phytozome.jgi.doe.gov/ (accessed on 1 December 2025)) [24]. Potential *GmCDK* and *GmCyclin* family members were identified using BLASTP (https://blast.ncbi.nlm.nih.gov/Blast.cgi (accessed on 3 December 2025)). To verify conserved domains, all candidate proteins were analyzed with SMART (http://smart.embl-heidelberg.de/ (accessed on 5 December 2025)). Gene lengths for the confirmed candidates were extracted from the GFF3 annotation file of the *Glycine max* Wm82.a6.v1 reference genome. The physicochemical parameters of the encoded proteins were assessed using the Expasy ProtParam tool (https://web.expasy.org/protparam/ (accessed on 8 December 2025)) [25], while subcellular localization predictions were performed with Euk-mPLoc 2.0 (http://www.csbio.sjtu.edu.cn/bioinf/euk-multi-2/ (accessed on 29 December 2025)) [26].

### 2.2. Phylogenetic Analysis of GmCDK and GmCyclin Genes

Multiple sequence alignment of the identified GmCDK and GmCyclin proteins was conducted with the MUSCLE program. A neighbor-joining phylogenetic tree was then generated using MEGA 11 software [27], applying a p-distance model, pairwise deletion, and 1000 bootstrap replicates. For the GmCDK tree construction, reference protein sequences from *Oryza sativa*, *Zea mays*, and *Arabidopsis thaliana* were included. The phylogenetic relationships of the GmCyclin family were analyzed exclusively using the soybean gene sequences.

### 2.3. Gene Structure, Conserved Protein Motifs, Chromosomal Location and Gene Duplication of GmCDK and GmCyclin

The genomic architecture of the identified *GmCDK* and *GmCyclin* genes was characterized using a combination of tools. TBtools (v2.390) was used to illustrate exon-intron structures [28]. Conserved protein motifs were predicted with the MEME suite (http://meme-suite.org/index.html (accessed on 8 January 2026)), set to discover a maximum of 10 motifs [29]. Furthermore, chromosomal localization data extracted from the genome annotation file facilitated physical mapping and the identification of putative gene duplication events, both visualized within the TBtools platform. Duplication events in *GmCDK* and *GmCyclin* genes within the soybean genome were detected using the Multiple Collinearity Scan toolkit (MCScanX, TBtools v2.389) and visualized with the CIRCOS program (TBtools v2.389) [30,31].

### 2.4. Cis-Acting Element Analysis

The complete *Glycine max* genome sequence was obtained from the Phytozome database (version 13). For all identified *GmCDK* and *GmCyclin* genes, promoter regions were defined as the 2.0 kb sequences upstream of the annotated transcription start sites and subsequently extracted. Putative cis-acting regulatory elements within these promoter sequences were predicted using the PlantCARE online tool (http://bioinformatics.psb.ugent.be/webtools/plantcare/html/ (accessed on 8 January 2026)) [32]. The resulting element annotations were visualized using TBtools software.

### 2.5. Tissue Expression Pattern Analysis Based on RNA Sequencing Data

Tissue-specific expression patterns of *GmCDK* and *GmCyclin* genes were analyzed using publicly available RNA sequencing data retrieved from the Phytozome database [33]. Gene expression levels were quantified using the metric Fragments Per Kilobase of exon per Million mapped fragments (FPKM). A heatmap was constructed with TBtools software to visually represent the observed expression profiles across tissues.

### 2.6. Plant Materials, RNA Isolation, cDNA Synthesis, and qRT-PCR

To analyze expression during seed development, seeds from four field-grown cultivars (Zhonghuang13, Guanyun, Donghai, and Sudou18) were sampled at three developmental stages: early (R2, seed mass < 10 mg), middle (R4, 30–50 mg), and late (R6, 115–150 mg). All samples were also flash-frozen in liquid nitrogen and processed for RNA isolation. Three biological replicates were included for each sample group.

Total RNA was isolated using the RNApure Plant Kit (DNase I) (CWBIO, Taizhou, Jiangsu, China) and first-strand cDNA was synthesized from approximately 1 µg of total RNA using the HiScript 1st Strand cDNA Synthesis Kit (Vazyme, Nanjing, Jinagsu, China). qRT-PCR was performed with iTaq Universal SYBR Green Supermix (Bio-Rad, Hercules, CA, USA) on a Bio-Rad CFX Connect™ Real-Time PCR System. The soybean *Actin11* gene [34] served as the internal reference and the relative expression levels of *GmCDK* and *GmCyclin* genes were calculated using the 2^−ΔΔCT^ method [35]. Each sample was analyzed with three technical replicates across three independent biological experiments. Statistical significance was assessed by one-way ANOVA, with a *p* < 0.05 considered significant. Primers were synthesized by Sangon Biotech (Shanghai, China), and their sequences are listed in Table S1.

### 2.7. Protein Interactors Prediction

The protein sequence of all GmCDK and GmCyclin members were analyzed using the STRING database V12.0 (https://cn.string-db.org/,Search Tool for the Retrieval of Interacting Genes/Proteins) to predict potential interactions between CDKs and Cyclins, with a confidence threshold set to 0.7.

### 2.8. Bimolecular Fluorescence Complementation (BiFC) Assay for GmCDK-GmCyclin Interactions

To investigate protein–protein interactions, the coding sequences of ten selected *GmCDK* and *GmCyclin* genes (stop codons removed; Primers see Table S1) were amplified. These fragments were directionally cloned into BiFC vectors: GmCDK sequences were fused to the N-terminal fragment of YFP in the pXY-103 vector, while GmCyclin sequences were inserted into the C-terminal YFP fragment of the pXY-104 vector. All constructs were verified by sequencing before being transformed into Agrobacterium tumefaciens strain GV3101 via a freeze–heat shock method. Paired combinations of the Agrobacterium strains were co-infiltrated into leaves of 4-week-old Nicotiana benthamiana plants. After 48 h, reconstituted YFP fluorescence was detected using a laser scanning confocal microscope.

## 3. Results

### 3.1. Genome-Wide Identification and Phylogenetic Tree Analysis of GmCDK and GmCyclin Gene Family in Soybean

A genome-wide search for *CDK* and *Cyclin* family members was performed in the *Glycine max* reference genome (Wm82.a6.v1) through BLASTP (https://blast.ncbi.nlm.nih.gov/Blast.cgi (accessed on 3 December 2025)). Using the conserved PF00069 or PF07714 domain sequence from known CDK proteins and PF00134, PF02984, PF08613 domain sequence from known Cyclin proteins in *Arabidopsis thaliana* as a query, a total of 28 and 101 non-redundant putative *GmCDK* and *GmCyclin* genes were identified, respectively. These identified genes were named based on their homology with the Arabidopsis homolog genes. The number of *GmCDK* and *GmCyclin* genes in soybean is much higher than that found in *Arabidopsis thaliana* (13/49), *Oryza sativa* (15/45), and *Zea mays* (16/53), suggesting possible evolutionary insight, e.g., expansion of this family in legumes.

**Figure 1:**
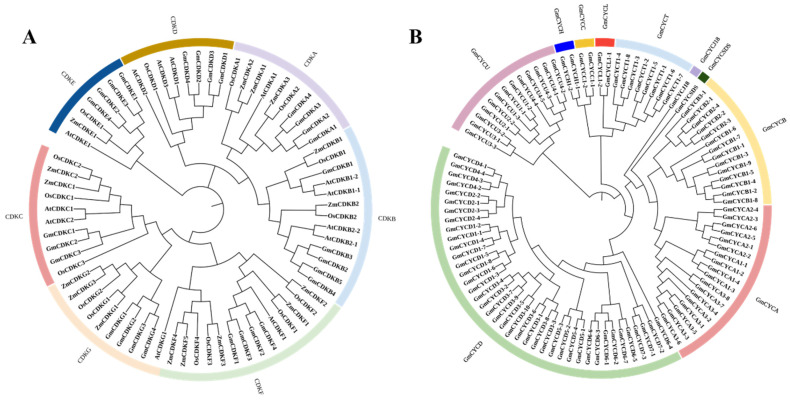
The phylogenetic tree of CDK and GmCyclin proteins. (**A**) Phylogenetic tree of CDK proteins from *Arabidopsis thaliana* (At), *Oryza sativa* (Os), *Zea mays* (Zm), and *Glycine max* (Gm). CDK proteins are classified into seven distinct subgroups, each highlighted with a different color: CDKA (purple red), CDKB (light blue), CDKC (pink), CDKD (orange), CDKE (dark blue), CDKF (light green), and CDKG (bisque). (**B**) Phylogenetic tree of GmCyclin proteins in soybean. GmCyclins are divided into ten subgroups, color-coded as follows: GmCYCA (pink), GmCYCB (yellow), GmCYCC (orange), GmCYCD (light green), GmCYCH (cyan), GmCYCT (light blue), CYCSDS (dark green), CYCU (violet), CYCJ18 (purple), and CYCL (red). These proteins were phylogenetically analyzed using MEGA11 software with 1000 bootstrap tests.

To elucidate the evolutionary relationships, a multiple sequence alignment of the full-length CDK protein sequences from soybean and representative species (*A*. *thaliana*, *O*. *sativa*, *Z*. *mays*) was performed. An unrooted phylogenetic tree was constructed using the neighbor-joining method with 1000 bootstrap replicates. The phylogenetic tree revealed that the CDK proteins could be classified into seven distinct major clades (CDKA to CDKG) (Figure 1A). All soybean CDK proteins were distributed across all seven clades, indicating the diversification of this family preceded the speciation of soybean. The phylogenetic tree revealed that Cyclin proteins could be classified into 10 distinct major clades (CYCA, CYCB, CYCC, CYCD, CYCH, CYCL, CYCT, CYCU, CYCJ18 and CYCSDS) (Figure 1B). GmCYCA and GmCYCD contained the largest number of members, suggesting these groups may have undergone significant expansion. Notably, GmCYCSDS and GmCYCJ18 consisted exclusively of proteins.

### 3.2. Chromosome Location, Duplication Events and Synteny Analysis of Soybean Genes Encoding CDK and Cyclin Proteins

**Figure 2:**
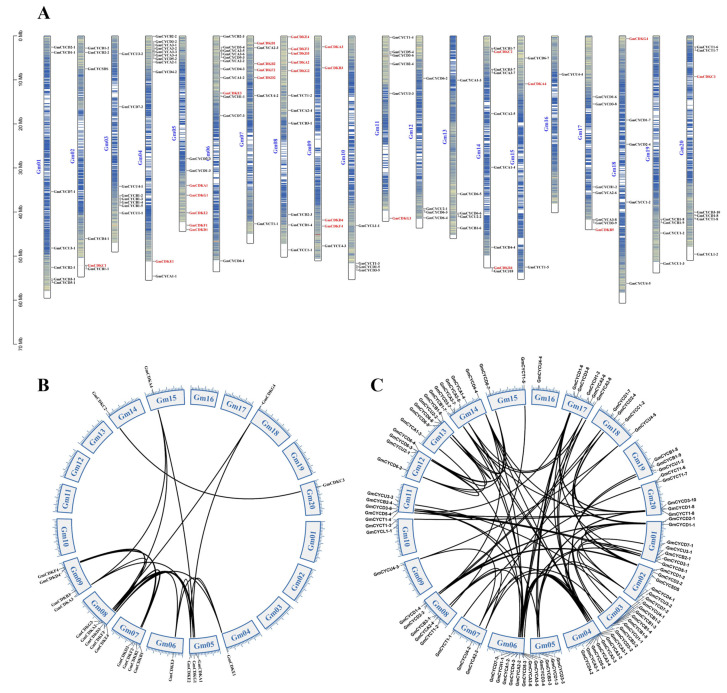
Chromosomal distribution and duplication events of *GmCDK* and *GmCyclin* genes in soybean. (**A**) Chromosomal localization of *GmCDK* and *GmCyclin* genes across the 20 soybean chromosomes. Chromosome size is indicated by its relative length. The scale bar is shown on the left, and the numbers represent the physical position of the genes on the chromosomes. The *GmCDK* and *GmCYC* genes are represented by red and black fonts respectively. (**B**) Duplication events among *GmCDK* genes. Black lines indicate *GmCDK* genes duplication events. (**C**) Duplication events among *GmCyclin* genes. Black lines indicate *GmCyclin* genes duplication events. Chromosomal locations and duplication events were analyzed using MCScanX (v2.389) and visualized with TBtools (v2.389).

To investigate the chromosomal distribution patterns of *GmCDK* and *GmCyclin* genes, each gene was systematically mapped to its corresponding chromosome using positional information from the soybean genome database. The analysis revealed that the 101 identified *GmCyclin* genes were unevenly distributed across all 20 chromosomes. Chromosome 6 harbored the highest number (11 genes), while chromosomes 9 and 16 each contained only one *GmCyclin* gene. Similarly, the 28 *GmCDK* genes were dispersed unevenly across 13 chromosomes, with the number of genes per chromosome ranging from one to five, indicating a non-uniform distribution across the genome (Figure 2A).

To explore the expansion mechanisms of these gene families in *G*. *max*, a duplication analysis was performed and visualized using Circos (TBtools v2.389). The results indicated that 22 *GmCDK* genes and 85 *GmCyclin* genes originated from whole-genome duplication (WGD) or segmental duplication events (Figure 2B,C). Additionally, tandem duplication events were identified among several *GmCyclin* genes, including *GmCYCA3-4*, *GmCYCB1-3*, *GmCYCB1-4*, *GmCYCB1-5*, *GmCYCB1-9*, and *GmCYCT1-7*. These findings suggest that WGD and segmental duplication have been the primary drivers of the *GmCDK* and *GmCyclin* gene family expansion, while tandem duplications contributed to localized gene clustering. In addition, the vast majority of orthologous gene pairs in both the *GmCDK* and *GmCyclin* gene families exhibited Ka/Ks ratios significantly less than 1, indicating strong purifying selection acting on these core cellular processes (Table S2).

**Figure 3:**
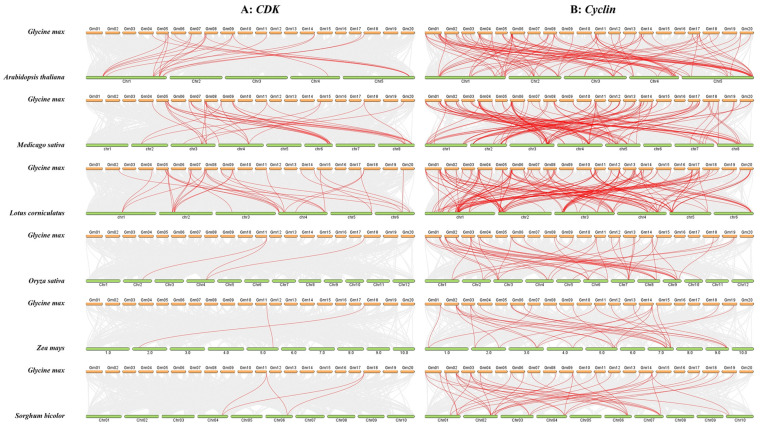
Collinearity of the *GmCDK* (**A**) and *GmCyclin* (**B**) gene pairs in soybean. Genes are listed in the outside of circle according to their chromosomal location. Synteny relationships between gene pairs are marked with a red line.

To further elucidate the evolutionary history of these genes, synteny analysis was conducted between soybean and three monocots (rice, maize, and sorghum), as well as three dicots (Arabidopsis, Medicago, and Lotus) (Figure 3). The results demonstrated extensive syntenic conservation of *GmCDK* and *GmCyclin* genes with the three dicot species. Soybean exhibited the highest degree of synteny with Medicago (31 *CDK* and 113 *Cyclin* syntenic pairs) and Lotus (32 *CDK* and 141 *Cyclin* syntenic pairs) across multiple chromosomes. A lower yet notable number of syntenic pairs were detected with Arabidopsis (20 *CDK* and 95 *Cyclin* pairs). In contrast, syntenic relationships were considerably fewer between soybean and the three monocots: rice (3 *CDK* and 43 *Cyclin* pairs), maize (2 *CDK* and 30 *Cyclin* pairs), and sorghum (4 *CDK* and 50 *Cyclin* pairs). Overall, syntenic gene pairs within the *CDK* and *Cyclin* families are significantly fewer in monocot crops than in dicot crops.

### 3.3. Basic Physicochemical Properties of the GmCDK and GmCyclin Proteins

To gain initial insights into the potential functional diversity of the *GmCDK* and *GmCyclin* family members, we first analyzed their basic physicochemical properties and predicted their subcellular localizations (Table S3). Sequence analysis indicates that the predicted molecular weights of GmCDK proteins range from 15.59 to 83.69 kDa, while those of GmCyclin proteins range from 14.07 to 81.75 kDa. Both the CDK and GmCyclin families exhibit a relatively broad isoelectric point (pI) distribution, spanning from 4.3 to 9.97. Proteins within the GmCDKA and GmCDKB subfamilies display relatively high stability, whereas those in the GmCDKC, GmCDKD GmCDKE, GmCDKF, and GmCDKG subfamilies show lower stability. An exception is GmCDKE3, which exhibits relatively high stability. In contrast, the stability coefficients for the vast majority of GmCyclin proteins exceed 40, suggesting that these may function as regulatory molecules with rapid turnover and short lifespans. The aliphatic index for most GmCDK and GmCYC family proteins falls between 70 and 100. However, GmCDKC1, GmCDKC2, GmCDKC3, GmCYCL1-1, and GmCYCL1-2 exhibit values around 60, while GmCYCB1-4 and GmCYCB1-5 exceed 110, indicating lower or higher thermal stability compared to other Cyclins. Based on the Grand Average of Hydropathicity (GRAVY), all GmCDK members are classified as hydrophilic proteins. The majority of GmCyclin proteins are also hydrophilic, with 16 exceptions identified as hydrophobic; for example, GmCYCJ18 is a hydrophobic protein. Subcellular localization predictions suggest that most GmCyclin proteins are nuclear, except for the CYCU subfamily, which is localized to both the cytoplasm and nucleus. Most GmCDK proteins are predicted to be present in both the nucleus and the cytoplasm.

### 3.4. Gene Structure and Conserved Amino Acid Motif Analysis of the CDK and Cyclin in Soybean

**Figure 4:**
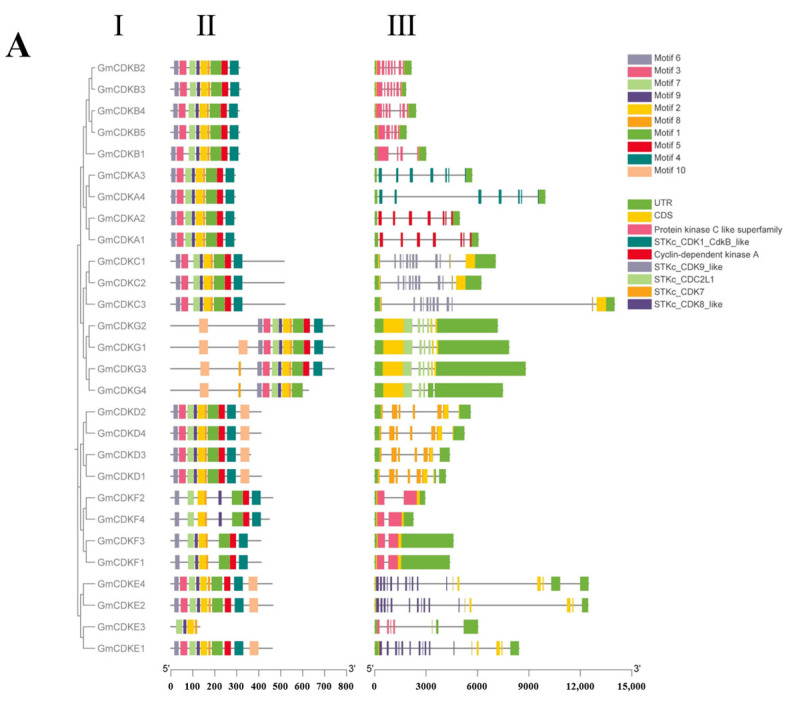

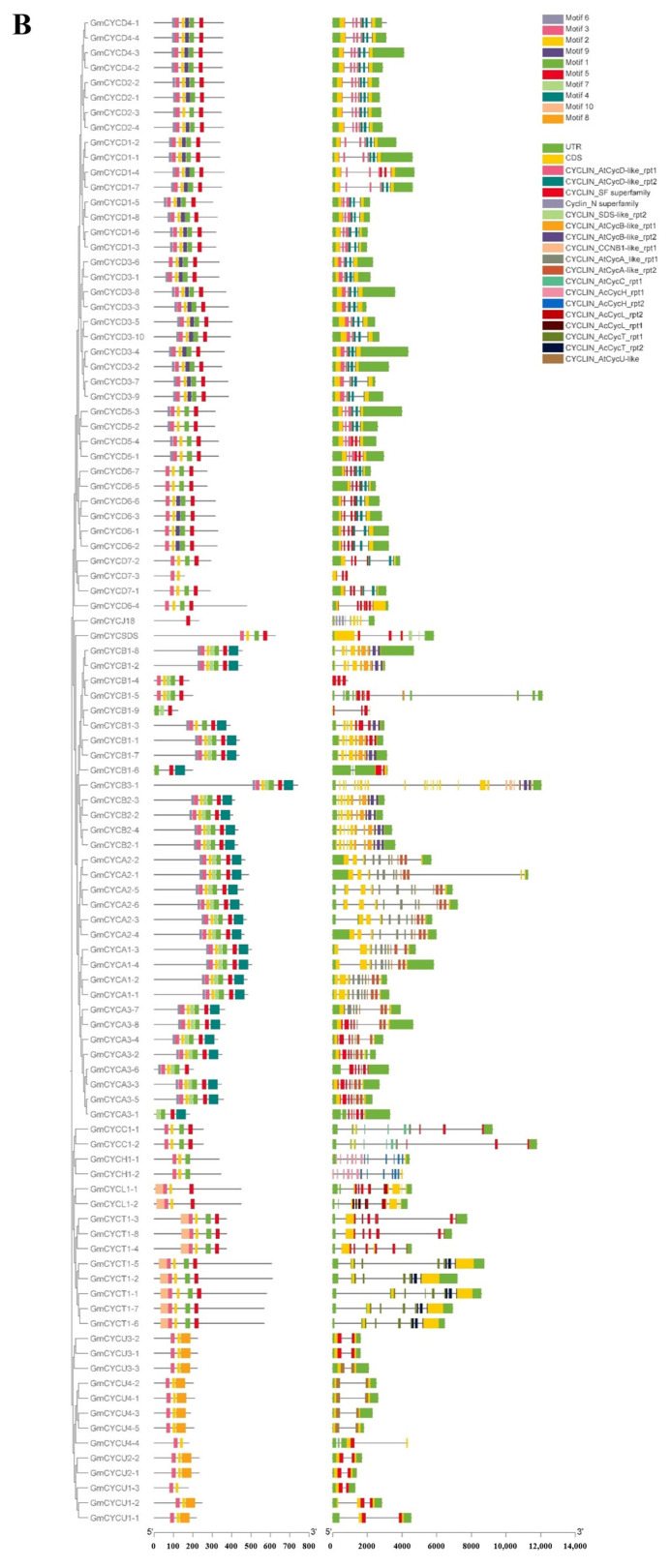
The phylogenetic relationships, gene structure, and motif compositions of GmCDK (**A**) and GmCyclin (**B**) genes in soybean. (**I**) 28 GmCDK and 101 GmCyclin proteins sequence was aligned using MUSCLE program and a neighbor-joining (NJ) tree was constructed using MEGA11 with 1000 bootstrap replicates, respectively; (**II**) The conserved motif distribution in each *GmCDK* or G*mCyclin* gene; (**III**) The location of CDK or Cyclin conserved domain and the length and patterns of exons, introns, and UTRs in each *GmCDK* or *GmCyclin* genes. The scales at the bottom of the image indicate the estimated exon/intron length in bp and motif length in numbers of amino acids (aa).

To elucidate the differences in gene structures, we conducted an in-depth analysis of the exons and introns within the *GmCDK* and *GmCyclin* gene sequences. Our findings revealed that, except for *GmCDKE*, which harbors only two exons, all other *GmCDK* genes possess more than four exons (Figure 4A,B). Similarly, the majority of *GmCYC* genes also contain more than four exons, with specific subgroups such as *GmCYCA* and *GmCYCB* exhibiting over ten exons each. In contrast, the *GmCYCU* subgroup is characterized by a notably simpler structure, containing only two exons.

To explore the conservation of functional domains within these genes, we performed a conserved motif analysis across all GmCDK and GmCyclin proteins (Figure 4A,B). In total, 10 motifs were detected using the MEME online website. Notably, all GmCDK, except for GmCDKE3 which contains only four motifs, were found to harbor between eight and ten motifs. On the other hand, all GmCyclin contained at least two motifs, with the highest number of motifs (seven) being detected in the GmCYCA and GmCYCB subgroups. The conserved domain analysis revealed that all GmCDK proteins contain a conserved CDK domain. In contrast, most GmCyclin proteins harbor two cyclin domains, except for CYCB1-6, CYCB1-9, CYCD7-3, CYCJ18, and members of the CYCU subfamily.

Moreover, our results highlighted a striking similarity in genetic structures among genes within the same subfamily. This similarity extends to the conserved motifs, where genes grouped together through phylogenetic tree analysis predominantly share identical motifs. This pattern of motif conservation not only reinforces the evolutionary relationships inferred from the phylogenetic analysis but also suggests functional conservation among closely related genes within the same subfamily.

### 3.5. Cis-Regulatory Element Analysis in the Promoter Regions of CDK and Cyclin Gene Families

**Figure 5:**
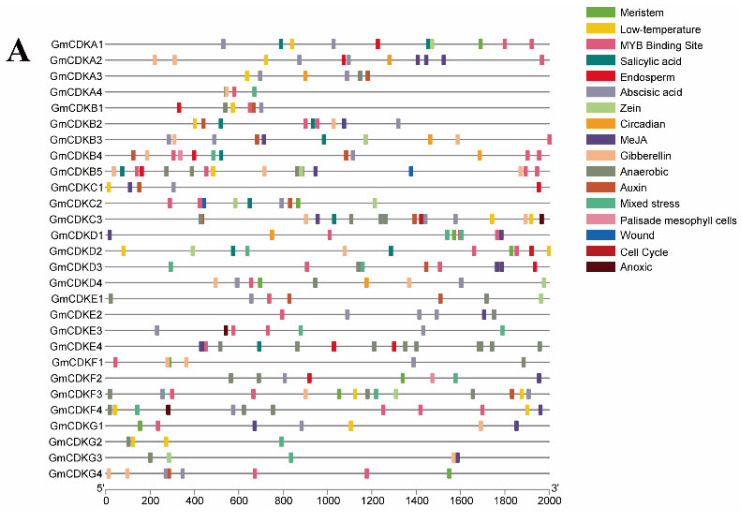

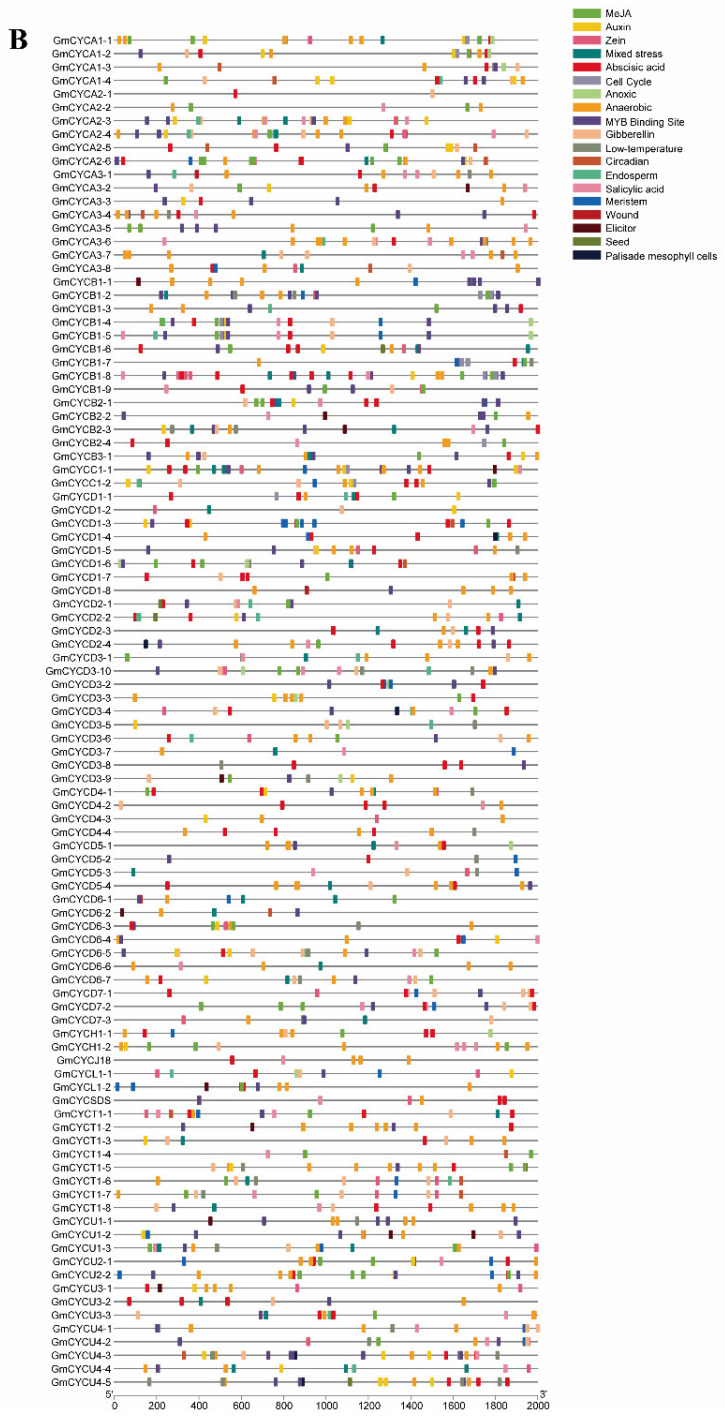
Predicted cis-elements in promoter regions of *GmCDK* (**A**) and *GmCyclin* (**B**) genes. The promoter region was defined as a 2 kb sequence upstream of the translation initiation codon of the gene. Identification of cis-acting elements using the online tool PlantCARE (https://bioinformatics.psb.ugent.be/webtools/plantcare/html/, (accessed on 3 December 2025)). Different types of cis-acting elements are represented by closed boxes of different colors.

To further investigate the biological functions of GmCDK and GmCyclin, the 2.0 kb upstream promoter sequences of all corresponding genes were retrieved and analyzed for cis-regulatory elements using the PlantCARE database. A diverse array of cis-regulatory elements was identified within the promoter regions of *GmCDK* and *GmCyclin* genes in *Glycine max* (Figure 5). These cis-acting elements, which serve as transcription factor binding sites, are critical for the regulation of gene expression. The composition and distribution of these elements varied among different genes.

In addition to the core promoter elements, CAAT-box and TATA-box, numerous cis-acting elements associated with development, phytohormone response, stress adaptation, cell cycle control, and tissue-specific expression were predicted. Multiple phytohormone-responsive elements were identified, including abscisic acid response elements (ABRE), methyl jasmonate-responsive motifs (CGTCA-motif and TGACG-motif), gibberellin-responsive elements (P-box, TATC-box, and GARE-motif), auxin-responsive elements (AuxRR-core and TGA-element), and salicylic acid-responsive elements (TCA-element and SARE), indicating that the expression of *GmCDK* and *GmCyclin* genes is likely modulated by various phytohormonal signals. Several stress-related elements were also detected, such as ARE (anaerobic induction element), GC-motif (anoxic induction element), TC-rich repeats (defense and stress response element), LTR (low-temperature responsiveness), and WUN-motif (wound responsiveness). Furthermore, elements involved in growth and development were predicted, including the endosperm expression-related GCN4-motif, meristem-specific CAT-box, Zein-responsive O2-site, palisade mesophyll cell-specific HD-Zip 1 binding site, and circadian/cell cycle-associated MSA-like motif. Additionally, multiple MYB transcription factor binding sites (CCAAT-box, MRE, MBS) were present in both *GmCDK* and *GmCyclin* gene families. Notably, seed-specific RY-element and elicitor-responsive AT-rich sequences were exclusively identified in GmCyclin promoters. These findings collectively suggest that cell cycle regulation in soybean is intricately linked with plant developmental processes as well as responses to environmental stimuli.

### 3.6. RNA Sequence Expression Pattern in Different Tissues in Soybean

**Figure 6:**
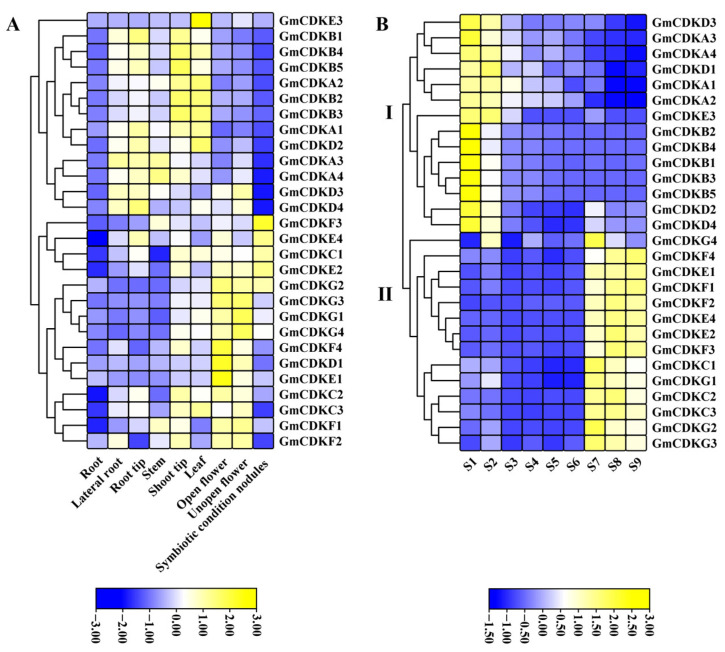

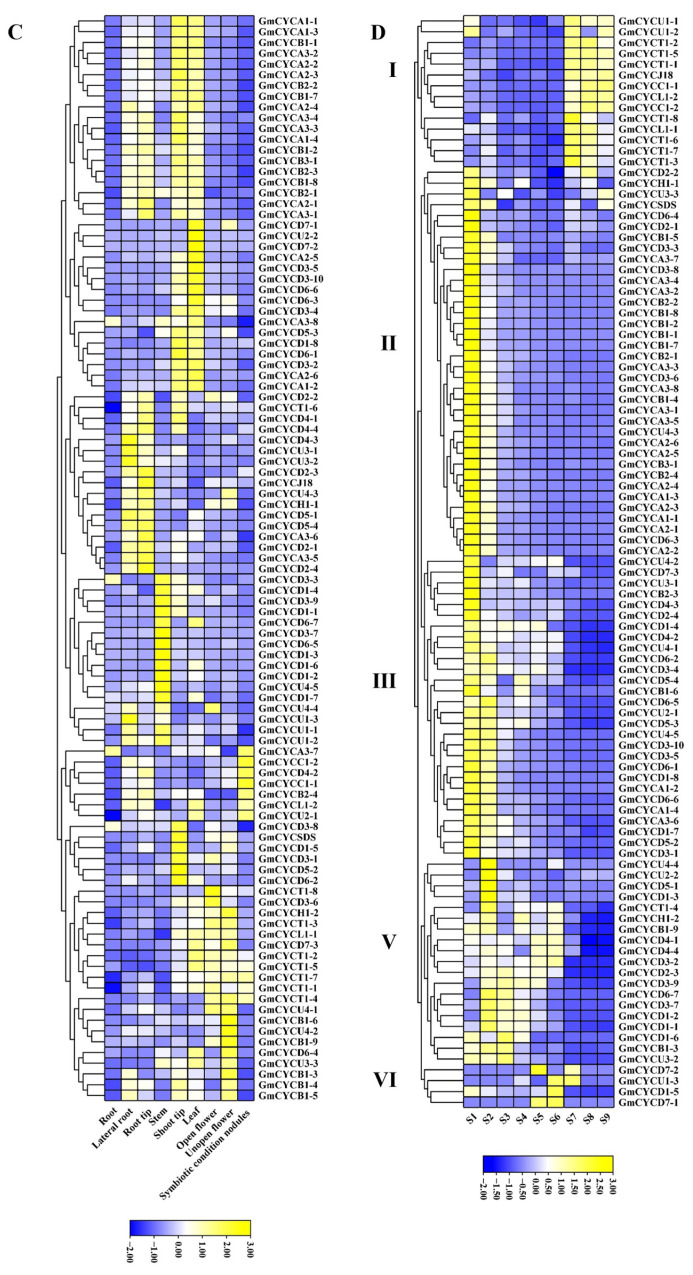
The expression profile of *GmCDK* (**A**,**B**) and *GmCyclin* (**C**,**D**) genes in different tissues. The color scale represents Log_2_(FPKM) values, where blue and yellow indicate low and high expression levels, respectively. The FPKM values of *GmCDK* and *GmCyclin* genes can be found in Table S4. A range of −3.00 (−2.00 or −1.50) to 3.00 was artificially set with the color scale limits according to the normalized values in the row. The expression profiles are categorized according to the values. GmCDK and GmCyclin formed two and five distinct clusters based on seed-stage expression heatmaps, respectively. The color scale shows increasing expression levels from blue to yellow. Flower tissue was collected from the opened flowers that had grown in the field in the flowering stage. Root, lateral root, root tip, shoot tip, leaf, and stem tissues were collected from 4-week-old plants grown on the B&D medium. The seed stage is based on the weight ranges as follows: S1 < 10 mg; S2, 30–50 mg (storage cells have large central vacuoles); S3, 70–90 mg (storage protein accumulation has begun, and subdivision of the vacuole is occurring); S4, 115–150 mg; S5, 200–250 mg (filling of the storage vacuoles); S6, >300 mg (green-colored seeds); S7, >300 mg (yellow-colored seeds); S8, 200–250 mg (fully mature, yellow-colored, and dehydrating seeds); and S9 < 150 mg (yellow-colored seeds and fully dehydrated).

In G. max, the expression profiles of *GmCDK* and *GmCyclin* genes were examined via RNA-seq across multiple organs—including root, lateral root, root tip, stem, shoot tip, leaf, open and unopened flowers, and nodules under symbiotic conditions—as well as across nine seed developmental stages (seed 1 to seed 9). As shown in Figure 6A, *CDK* gene expression varied substantially among tissues. *GmCDKA*, *GmCDKB*, and *GmCDKC* genes were highly expressed in leaves, whereas *GmCDKC* and *GmCDKF* were notably higher in root nodules. Shoot tips exhibited high expression of *GmCDKB2*, *GmCDKA2*, *GmCYCA3-7*, *GmCYCC1-1*, *GmCYCC1-2*, and *GmCYCD4-2*. Certain *Cyclin* genes, including *GmCYCD7-1*, *GmCYCU2-2*, and *GmCYCD7-2*, showed leaf-specific high expression (Figure 6C).

Based on seed-stage expression heatmaps (Figure 6B,D), *GmCDK* genes formed two distinct clusters, each characterized by similar expression patterns (Group A,B, Figure 6B). Genes in Group I exhibited high expression during the early stages of seed development (seed 1–2), whereas those in Group II showed elevated expression in later stages (seed 7–9). *GmCyclin* genes segregated into five expression clusters (Group I–V, Figure 6D). Compared with other developmental periods, Group I genes were more highly expressed during late-stage development (seed 7–9). Group II genes displayed higher expression in early-stage development (seed 1–2). Group III genes were highly expressed in the early stages and maintained moderate expression during seed 2–6. Group IV genes were primarily expressed in mid-development (seed 2–6), while Group V genes were mainly active from mid- to late-development stages (seed 5–7).

### 3.7. qRT-PCR Analysis of CDK and Cyclin Genes at Three Different Seed Development Stages Among Four Different Germplasms of Soybeans

**Figure 7:**
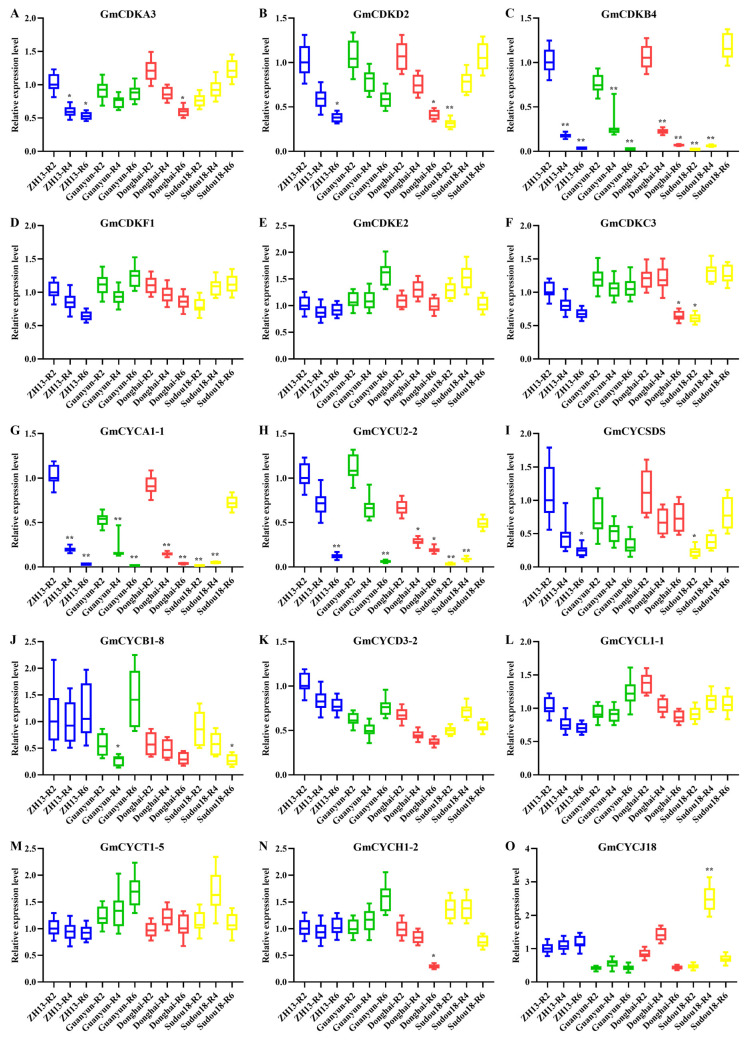
Temporal expression profiles of six *GmCDK* (**A**–**F**) and nine *GmCyclin* (**G**–**O**) genes as measured by qRT-PCR in four different soybean varieties. Four soybean varieties: ZH13 (blue), Guanyun (green), Donghai (red) and Sudou13 (yellow). R2, 30–50 mg (storage cells have large central vacuoles); R4, 115–150 mg; R6, >300 mg (green-colored seeds). Data are expressed as mean ± SD (*n* = 3 independent assays). ** and * indicate significant differences compared to the control (ZH13-S2) at *p* < 0.01, and *p* < 0.05, respectively.

To ensure a comprehensive yet feasible experimental validation, we deliberately selected one representative gene from each major subfamily based on the phylogenetic trees constructed in our study. This strategy guaranteed that our RT-qPCR results could reflect the transcriptional dynamics of the entire gene family rather than being biased toward a single subfamily. Quantitative analysis of mRNA expression levels for six *GmCDK* and nine *GmCyclin* genes was conducted across three seed developmental stages in four soybean germplasms: ZH13, Guanyun, Donghai, and Sudou18. *GmACTIN* served as the internal reference gene for normalization. In Zhonghuang13, Guanyun, and Donghai, transcript levels of *GmCDKA3*, *GmCDKD2*, and *GmCDKB4* generally decreased as seed development progressed (Figure 7A,C). In contrast, in Sudou18, expression of these three genes increased during maturation (Figure 7A,C). Notably, *GmCDKB4* expression was significantly downregulated at the R4 and R6 stages in ZH13, Guanyun, and Donghai but markedly upregulated in Sudou18 (Figure 7C). Expression of *GmCDKF1* and *GmCDKE2* remained relatively stable across developmental stages and among the four varieties (Figure 7D,E). *GmCDKC3* expression did not differ significantly between ZH13 and Guanyun; however, it was lower at the R6 stage in Donghai and at the R2 stage in Sudou18 compared to other stages within the same variety (Figure 7F).

Within the *GmCYC* gene family, expression of *GmCYCA1-1*, *GmCYCU2-2*, and *GmCYCSDS* declined during seed development in three of the four varieties, while increasing in Sudou18 (Figure 7G–I). In Guanyun, transcript levels of *GmCYCB1-8*, *GmCYCD3-2*, *GmCYCL1-1* and *GmCYCT1-5* showed a moderate increase during development, whereas the other three varieties exhibited either no significant change or slight downregulation (Figure 7J–M). *GmCYCH1-2* expression was significantly reduced at the R6 stage in Donghai and Sudou18 but elevated at the same stage in Guanyun (Figure 7N). Additionally, *GmCYCJ18* expression was downregulated at both the R4 and R6 stages in Sudou18, with no notable changes observed in the remaining varieties (Figure 7O). These results suggest that distinct *CDK* and *Cyclin* genes are differentially regulated during seed development.

### 3.8. CDK and Cyclin Protein Interaction Network Construction

**Figure 8:**
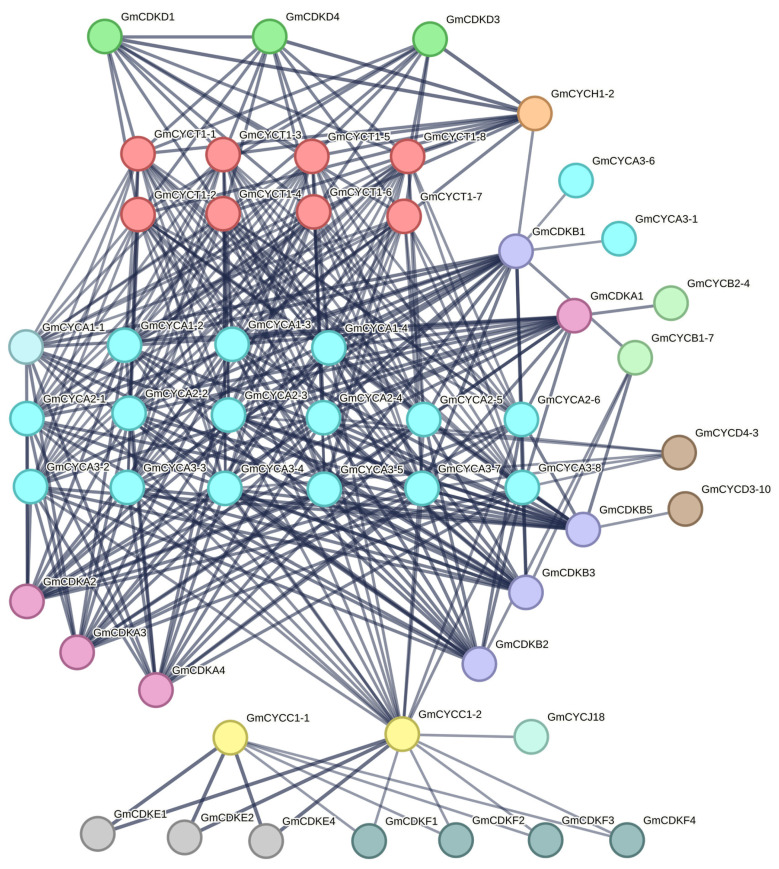
GmCDK-GmCyclin protein network diagram visualized by STRING. The interaction network was generated using the STRING database (version 12.0) (https://string-db.org/). The network includes proteins resulting from the 28 GmCDK and 101 GmCyclin proteins. Only interactions with a combined confidence score >0.70 are displayed. Each node represents a protein, and each edge represents a predicted or known functional association. Proteins belonging to the same subfamily are represented by the same color. Edges are showing by confidence (line thickness indicates the strength of data support).

To explore potential interactions between GmCDK and GmCyclin protein families, a protein–protein interaction (PPI) network was predicted using the STRING database. As shown in Figure 8, the GmCDKA and GmCDKB subgroups are predicted to interact with the GmCYCA1, GmCYCA2, and GmCYCA3 subgroups, respectively. The GmCDKD subgroup interacts with the CYCT subgroup and with CYCH1-2, whereas GmCDKE and GmCDKF interact with CYCC. Additionally, CYCB1-7 is predicted to interact with CDKB1, CDKB2, CDKB3, and CDKB5; CYCD3-10 interacts exclusively with CDKB5; and CYCB2-4 interacts only with CDKA1.

**Figure 9:**
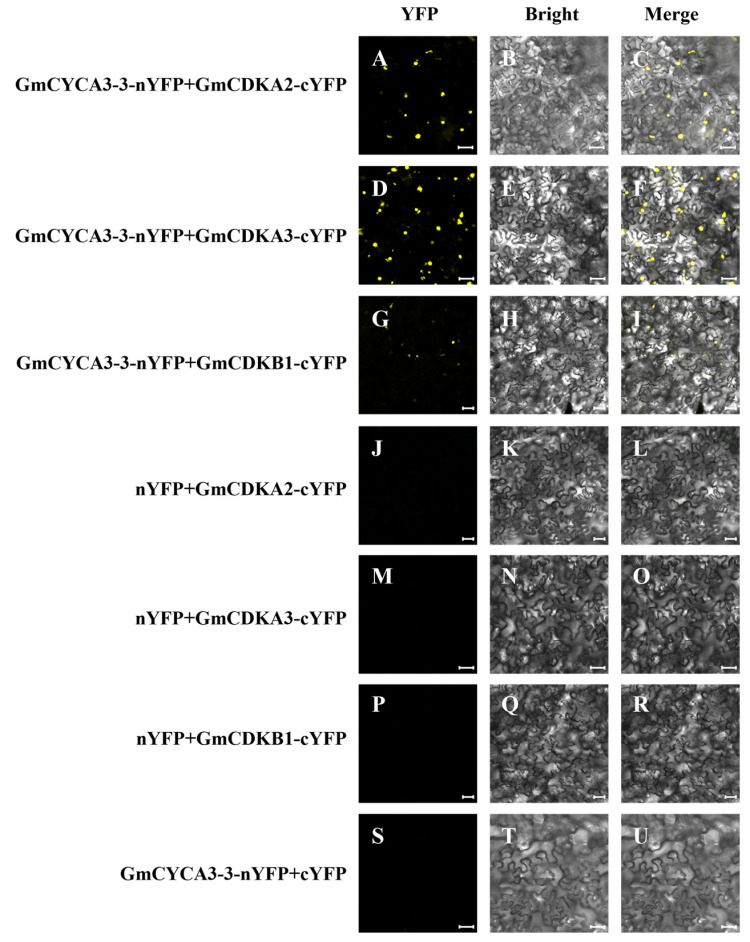
BiFC analysis of the interaction between GmCDK and GmCYC in *Nicotiana benthamiana* epidermal cells. Representative confocal images show YFP fluorescence signals, indicating specific interaction between GmCDKA2 and GmCYCA3-3 (**A**–**C**), between GmCDKA3 and GmCYCA3-3 (**D**–**F**) and between GmCDKB1 and GmCYCA3-3 (**G**–**I**). Negative controls (**J**–**U**), co-expressing empty vectors, exhibited no detectable fluorescence. Scale bars: 50 µm. All images were taken at 48 h post-agroinfiltration using a confocal microscope (Zeiss LSM 880) with a 10× objective.

Based on this PPI network, several key core cell cycle proteins were selected for further experimental validation in soybean using BiFC assays. These included eight GmCDK proteins (GmCDKA1, GmCDKA2, GmCDKA3, GmCDKA4, GmCDKB1, GmCDKB4, GmCDKB5, and GmCDKF2) and two GmCYC proteins (GmCYC1-4 and GmCYC3-3). Among these, only GmCDKA2, GmCDKA3 and GmCDKB1 showed a positive interaction signal with GmCYCA3-3 (Figure 9). We further examined the expression patterns of these genes across different tissues. GmCDKA2, GmCDKA3 and GmCDKB1 showed similar tissue expression pattern with a high expression level in lateral roots, root tips, stems, and shoot tips. In addition, GmCDKA2 was highly expressed in shoot tips, root tips, leaves, and stage S1 (Figure 5). These overlapping expression profiles support their potential role in active cell division processes in plants.

## 4. Discussion

A genome-wide analysis of the soybean (*Glycine max*) genome identified 28 *GmCDK* and 101 *GmCyclin* genes, establishing a comprehensive profile of these core cell cycle regulators (Table S2). Phylogenetic analysis classified the *GmCDKs* into the seven canonical plant clades (CDKA-CDKG), a structure conserved across species. This indicates that the fundamental functional diversification of the *CDK* family was established prior to the divergence of major plant lineages. The representation of soybean genes in all seven clades further underscores the deep conservation of the core cell cycle machinery. In contrast, the *Cyclin* family exhibits greater complexity and lineage-specific diversification. The 101 *GmCyclin* genes were classified into ten subfamilies (A-, B-, C-, D-, H-, L-, T-, U-, SDS-, and J18-type). While these ten subtypes are present in *Arabidopsis thaliana* [36], *Medicago truncatula* [37], and tomato [38]. The J18-type is absent in *Brassica rapa* [39] and rice [40]. The J18-, H- and U-types are also missing in maize [21]. Notably, an F-type cyclin has been detected specifically in rice and maize [21,40]. Further underscoring this diversity, *Marchantia polymorpha* possesses only eight *Cyclin* genes with only one or two copies, indicating minimal redundancy within its *Cyclin* gene family [41]. The pronounced divergence in *Cyclin* gene composition between complex angiosperms like soybean and early-diverging plants such as liverworts provides valuable insights into plant evolutionary mechanisms. The substantial expansion of the *Cyclin* gene family in soybean likely reflects evolutionary adaptations tied to its specialized developmental programs, including intricate nodulation processes and seed development.

The analysis of gene structure and conserved motifs provides robust complementary evidence to the phylogenetic classification and reinforces the principles of evolutionary conservation and functional divergence within the *GmCDK* and *GmCyclin* gene families. The general trend of complex gene structures, particularly the high number of exons observed in most *GmCDK* genes and in key subfamilies like *GmCYCA* and *GmCYCB*, is consistent with their roles as core regulatory components (Figure 4). Complex exon-intron architecture can facilitate alternative splicing, a potential mechanism for generating protein diversity and fine-tuning regulation in response to developmental or environmental cues [42]. The variation in motif number, particularly the reduced motifs in GmCDKE3, CYCB1-6, CYCB1-9, CYCD7-3, CYCJ18, and the entire CYCU subfamily, may indicate structural adaptations or the loss of regulatory segments, potentially fine-tuning its interaction specificity or regulation (Figure 1B and Figure 4B). Compared with PhvulCycU subfamily, GmCYCU4-4 and GmCYCU1-3 do not contain the conserved motif [37]. Future functional studies should prioritize these divergent members to uncover their unique roles to reveal novel cell cycle-related regulatory mechanisms in soybean.

Our findings robustly demonstrate that WGD or segmental duplication has been the predominant force behind the expansion of both gene families. The high proportion of genes originating from such events (22 of 28 *GmCDKs* and 85 of 101 *GmCyclins*) aligns perfectly with the known paleopolyploid history of the soybean genome [43]. The comparative synteny analysis indicated that the *CDK* and *Cyclin* gene families underwent substantial lineage-specific reorganization following their evolutionary split. The limited syntenic correspondence with monocots suggests that, while the core cell cycle machinery is functionally conserved, its genomic context has been significantly reshaped by lineage-specific duplications, losses, or chromosomal rearrangements.

The broad molecular weight ranges of GmCDKs and GmCyclins (~15–84 kDa) reflect their functional diversification, which likely stems from the acquisition of diverse N- or C-terminal regulatory domains that fine-tune protein interactions, substrate specificity, and stability. It was reported that CDKA and CDKB proteins serve as core cell cycle regulators in plants, controlling key transitions such as G1/S and G2/M [44]. Consistent with their constitutive catalytic roles, GmCDKA and GmCDKB subfamilies exhibit relatively high stability. In contrast, cyclins function as transient activators whose precise, timed degradation—mediated by the ubiquitin-proteasome system—is essential for orderly cell cycle progression. Accordingly, most GmCyclin proteins display a high instability index (>40), aligning with their regulated turnover. Variations in the aliphatic index and GRAVY values provide clues about subfamily-specific adaptations. For example, the exceptionally high values (>110) for GmCYCB1-4 and GmCYCB1-5 imply increased thermal stability, which may be crucial for their activity during the intense and rapid processes of mitosis, where these B-type cyclins are key regulators.

Promoter cis-element analysis provides crucial regulatory clues for understanding the complex spatiotemporal expression patterns of *GmCDK* and *GmCyclin* genes. The promoters of these genes not only contain basic transcriptional elements but also are enriched with elements that respond to various hormones such as ABA, GA, and JA. This is highly consistent with their dynamic expression at different stages of seed development, suggesting that the antagonism and synergy of hormone signals are the core switches for precisely regulating the transition of cell cycle stages [45]. It is worth noting that the specific seed development-related RY elements present in the GmCyclin promoter may explain the specific upregulation of some *Cyclin* genes during the grain filling stage (such as in Sudou18). It is suggested that the Cyclin subfamily plays a more dominant regulatory role in connecting seed-specific development programs with the cell cycle process [46]. Furthermore, the presence of many stress response elements places the cell cycle at the core of environmental perception, indicating that the stability of crop yield may partly depend on the ‘programmed’ response ability of the cell cycle machine to abiotic stress.

The transcriptome analysis of this study revealed the complex spatiotemporal expression profiles of the *GmCDK* and *GmCyclin* gene families in soybeans. In vegetative organs, the specific high expression of *GmCDKC* and *GmCDKF* in root nodules is particularly remarkable (Figure 6), suggesting that they may play a special role in establishing and maintaining an efficient symbiotic nitrogen fixation system by regulating the internal replication cycle. During seed development, gene expression exhibits clear stage modularization characteristics (Figure 6B,D). For instance, the Group B gene, which is highly expressed in the early stage, may drive the syncytic division of endosperm, while the Group A gene, which is active in the later stage, may be related to the initiation of maturity-related programs. The RT-qPCR results revealed substantial expression divergence of CDK and Cyclin genes among the four soybean germplasms during seed development (Figure 7). Notably, core mitotic regulators (*GmCDKA3*, *GmCDKB4*, *GmCYCA1-1*) showed opposite trajectories in Sudou18 versus the other varieties—sustained or increased expression during late maturation—suggesting prolonged cell division activity that may contribute to larger seed size. Additionally, *GmCYCD3-2* and *GmCYCL1-1* were specifically upregulated in Guanyun, hinting at variety-specific hormone signaling that fine-tunes seed filling. Among the candidates, *GmCDKB4*, *GmCYCD3-2*, and *GmCYCJ18* emerge as priority targets for functional validation via gene editing. These stage- and genotype-specific patterns provide a foundation for developing molecular markers associated with seed weight and offer actionable targets for breeding programs aimed at yield improvement in soybean. This phenomenon is different from the typical patterns of late expression inhibition of cell cycle genes reported in *Arabidopsis thaliana* and rice, and may reflect the specific domestication adaptation of soybean germplasm [46]. Further combination with promoter variation analysis or protein–protein interaction verification will help to understand how these genes integrate hormone signals (such as gibberellin) to regulate the final seed size, providing new targets for molecular design breeding.

Studies of the cell cycle core complex in *Arabidopsis thaliana* have clarified that mitotic A/B-type cyclins specifically bind B-type CDKs rather than CDKA;1, while D-type, S-type, and A-type cyclins preferentially interact with CDKA;1 [47]. This work revealed that plants utilize a diverse repertoire of CDK–Cyclin complexes as a regulatory toolkit, highlighting both the functional diversification of cyclins and the central role of cell cycle regulation in plant developmental plasticity [48]. Subsequent research has further elucidated specific functions of these complexes. For example, the CDKA/CYCD3 complex maintains the apical meristem by balancing stem cell proliferation and differentiation through E2F transcription factor regulation [44,49]. In rice, B2-type cyclins interact with B-type CDKs to regulate mitosis, underscoring the importance of plant-specific cyclin–CDK complexes in cell cycle control [50]. More recently, auxin-mediated leaf morphogenesis in white poplar was shown to involve specific D-type cyclins (PoalbCYCD1;4, PoalbCYCD3;3, and PoalbCYCD3;5) forming complexes with PoalbCDKA;1 to drive the G1–S transition [51]. Our BiFC assays revealed specific interaction of GmCDKA2, GmCDKA3, and GmCDKB1 with GmCYCA3-3, while other tested CDKs showed no signal (Figure 9). This selectivity is biologically informative: GmCYCA3-3 may act as a dual-purpose regulator engaging distinct CDK partners at different checkpoints, consistent with CDKA driving G1/S and CDKB governing G2/M [2,4,6]. The absence of interaction with other CDK paralogs suggests subfunctionalization following soybean whole-genome duplication, with duplicated copies acquiring specialized roles [46]. Co-expression of the three interacting partners in meristematic tissues (Figure 5) supports their functional relevance. The GmCDKA2/A3/B1–CYCA3-3 module likely represents a core complex driving mitotic proliferation. Future work should prioritize spatiotemporal mapping, CRISPR/Cas9 mutants, and binding affinity assays to validate its role in cell division and seed development, offering potential targets for improving legume crops.

## 5. Conclusions

This study systematically characterized the core cell cycle regulators in soybean, identifying 28 *GmCDK* and 101 *GmCyclin* genes. Their expansion primarily resulted from whole-genome duplication, with lineage-specific reorganization shaping the genome. Structural and promoter analyses revealed functional diversification and complex regulation by hormones and stress. Expression profiling highlighted roles in seed development. GmCDKA2, GmCDKA3 and GmCDKB1 can interact with GmCYCA3-3, respectively. These findings provide a foundation for future research to improve soybean yield and stress resistance.

## Data Availability

Data is available from the authors upon request.

## Supplementary Materials

The following supporting information can be downloaded at: https://www.mdpi.com/article/10.3390/biology15131065/s1, Table S1: Primers pairs used in this study; Table S2: Protein physiochemical properties of GmCDK and GmCyclin; Table S3: Complete Ka/Ks analysis of gene pair in soybean *CDK* and *Cyclin* gene family; Table S4: The expression profile of *GmCDK*(A, B) and *GmCyclin* (C, D) genes in different tissues.

## Abbreviations

The following abbreviations are used in this manuscript:

CDKs	cyclin-dependent kinases
RBR	retinoblastoma-related protein
WGD	whole-genome duplication
pI	isoelectric point
GRAVY	Grand Average of Hydropathicity
PPI	protein–protein interaction
BiFC	bimolecular fluorescence complementation assays
FPKM	Fragments Per Kilobase of exon per Million mapped fragments
